# Heated drinking water in winter improves growth performance of male Hu sheep by modulating rumen quorum sensing and metabolites, and enhancing serum antioxidant capacity

**DOI:** 10.5713/ab.24.0821

**Published:** 2025-04-04

**Authors:** Chang Liu, Lingyan Li, Jiaqi Dai, Mingren Qu, Kehui Ouyang, Qinghua Qiu

**Affiliations:** 1 Jiangxi Province Key Laboratory of Animal Nutrition and Feed, College of Animal Science and Technology, Jiangxi Agricultural University, Nanchang, China; 2 College of Animal Science and Veterinary Medicine, Heilongjiang Bayi Agricultural University, Daqing, China

**Keywords:** Antioxidant Capacity, Biofilm Formation, Drinking Water Temperature, Quorum Sensing, Rumen Fermentation Characteristic, Rumen Microbe

## Abstract

**Objective:**

This study aimed to explore the mechanism by which increasing the temperature of drinking water in winter promotes sheep growth from a microbiological perspective.

**Methods:**

A total of 12 healthy male Hu sheep were evenly divided into two groups: one with drinking water at 12°C (WT12) and the other at 25°C (WT25), and they were raised for 60 days in the cold winter.

**Results:**

The WT25 group had higher average daily gain, serum immunoglobulin G, total antioxidant capacity, glutathione peroxidase, and superoxide dismutase, along with lower feed-to-gain ratio, serum cortisol, malondialdehyde, reactive oxygen species, and oxidative stress index when compared to the WT12 group (p<0.05). The concentrations of microbial crude protein, microbial density, autoinducer-2 signaling molecule concentration, and biofilm formation were higher in the WT25 group, while the ammonia nitrogen concentration was lower (p<0.05). The relative abundances of *Muribaculum* and *Clostridia UCG-014*, as well as the predicted metabolic pathways related to lipid metabolism, were lower in the WT25 group, whereas the metabolism of other amino acids showed increased abundances (p<0.05). Both principal coordinates analysis and analysis of similarities revealed no significant differences in rumen microbial communities between the WT12 and WT25 groups (p>0.05). Metabolomics analysis identified 12 differential metabolites, four of which were correlated with *Muribaculum*, *Raoultibacter*, and Coriobacteriales Incertae Sedis.

**Conclusion:**

These findings suggest that heated drinking water in winter may improve growth performance by increasing rumen microbial biofilm formation and enhancing serum antioxidant capacity in Hu sheep. This study reveals links between rumen microbial quorum sensing and critical parameters such as animal growth phenotypes, rumen metabolic characteristics, and specific bacterial genera. It offers innovative perspectives on enhancing animal feed efficiency through the modulation of rumen microbial quorum sensing.

## INTRODUCTION

Ruminants possess a unique digestive system characterized by a specialized stomach compartment known as the rumen. This organ is instrumental in the breakdown of fibrous plant material, facilitated by a community of symbiotic microorganisms [1]. The rumen’s fermentation process not only breaks down cellulose into digestible compounds but also produces volatile fatty acids (VFA), which are critical for the animal’s energy metabolism [2]. Moreover, the rumen is indispensable for the biosynthesis of vital nutrients, such as amino acids and vitamins, highlighting its nutritional importance [3]. The rumen microbiome is a complex and dynamic ecosystem that comprises bacteria, archaea, protozoa, and fungi [4]. The balance and composition of the rumen microbiota directly influence the host’s ability to extract nutrients from feed, as well as its overall health and productivity [3]. Therefore, a thorough comprehension of the rumen functionality and microbiota community offers opportunities for developing strategies to optimize ruminant nutrition and health.

Temperature fluctuations have profound implications for ruminant health and rumen ecosystem. The rumen is a complex and delicate environment where temperature plays a crucial role in maintaining the metabolic activities of microorganisms responsible for digestion [5]. Temperature fluctuations can disrupt this balance, leading to a decline in rumen motility and altered fermentation patterns [5]. These detrimental changes can affect the overall health and productivity of the ruminants. The temperature of drinking water can also affect the rumen’s microenvironment, because water is not only a critical component of the rumen fluid but also plays a significant role in maintaining fermentation processes. Recent studies have found that the temperature of drinking water can significantly affect rumen fermentation function, serum antioxidant capacity, and overall animal growth performance, particularly in cold seasons [6]. Specifically, the concentrations of total VFA, propionate, and cellulase in rumen fluid, when drinking water was at 18.6°C, notably surpassed those at 4.39°C. Additionally, marked enhancements in the average daily gain (ADG), serum total antioxidant capacity (T-AOC), and apparent digestibility of neutral detergent fiber were observed [6]. This is consistent with earlier research which demonstrated that the use of heated drinking water is beneficial in decreasing the duration for which ruminal pH falls below the critical threshold and in elevating ruminal temperature [7]. Numerous studies on dairy cows and beef cattle have revealed the effects of heated drinking water temperature on rumen function, including its impact on rumen temperature, stability, and feed efficiency, which are influenced by water intake and feed consumption [8–10]. These effects from heated water collectively promote enhanced microbial activity and improved digestion of nutrients, ultimately resulting in an enhancement of growth performance. Cold water intake can lead to a rapid decrease in rumen temperature, potentially slowing down microbial activity and feed digestion [8]. Conversely, water that is too warm can lead to an overproduction of VFA and potentially result in acidosis if the rumen’s buffering capacity is exceeded [11]. It should be noted that the practice of heating drinking water can lead to increased investment in equipment and higher electricity expenses for the farm. Moreover, there is a risk that warmer water could foster algal blooms, leading to eutrophication and potentially harming animal health [12]. Therefore, the selection of optimal temperature for drinking water is crucial for maintaining the rumen’s homeostasis and ensuring efficient nutrient utilization, as well as achieving the best economic benefits in animal husbandry. Current studies predominantly suggest that the optimal temperature for drinking water in winter falls within the range of 20°C–30°C, depending on factors such as animal species, body weight, type of feed, and environmental temperature [6–8].

Quorum sensing (QS) is a sophisticated communication mechanism employed by bacteria to evaluate their population density, aligning their collective behavior in response to autoinducers, such as biofilm formation and bioluminescence [13]. This intricate system plays a vital role in the adaptation and survival of bacterial communities across various adverse environments [13]. Within rumen microbial communities, the LuxS/autoinducer-2 (AI-2) system stands out as a primary mode of QS [14,15]. The enzyme LuxS is responsible for generating AI-2, a signaling molecule that enables interspecies dialogue [14]. Water that is either too hot or too cold compared to the rumen temperature can be a stressor for rumen microbes. Based on the aforementioned theory, it is speculated that, in response to stress, rumen microbes might initiate QS to mitigate the stress. However, according to the current literature available to us, this hypothesis has not yet been reported. Therefore, it is essential to explore whether the enhancement of production performance by increasing water temperature is related to QS and to investigate the potential underlying mechanisms that rumen microbes might facilitate.

Therefore, a 60-day feeding trial was conducted to explore the effects of heated drinking water on the growth performance, serum biochemistry, rumen fermentation characteristics, microbial community, and rumen metabolites of male Hu sheep. The study aims to clarify the mechanism by which heated drinking water in winter promotes the growth performance of Hu sheep and to determine if QS is involved in this process. It is hypothesized that increasing the drinking water temperature in winter could induce QS, leading to increased biofilm formation, which may in turn enhance the serum antioxidant capacity, and ultimately promote the productive performance of ruminants. This study may broaden new perspectives on the mechanism by which heated drinking water promotes animal growth in winter, aiding in the application of precision feeding techniques for ruminant animals.

## MATERIALS AND METHODS

### Animals and experimental design

A total of 12 healthy male Hu sheep, with similar body weights (27.70±0.48 kg) and ages (141.15±0.87 days), were evenly divided into two groups, each consisting of six animals. One group was provided with clean water at a temperature of 12°C, while the other group received clean water at a temperature of 25°C. The control of water temperature was achieved using a thermostatic heating rod. The ingredient composition and nutritional content are detailed in Table 1. The entire experimental period lasted for 60 days, including a transition period of 15 days and an experimental period of 45 days. The average ambient temperature during the experiment was 10.63°C, and the humidity was 75.59%. The sheep were individually housed, allowed to feed freely at their own pace, and had access to fresh drinking water at all times.

### Sample collection

Feed intake was meticulously recorded daily for each sheep. Prior to the start of the experiment and upon its end, the sheep were weighed over two consecutive days. Blood samples were collected before morning feeding via jugular venipuncture into tubes without anticoagulants. These blood samples were then centrifuged at 2,012×g for 15 minutes to obtain the serum. Rumen contents were collected before morning feeding using the oral intubation technique as described by Paz et al [16], which includes both solid and liquid fractions. The rumen fluid, obtained by filtering rumen contents through four layers of cheesecloth, was reserved for further examination of rumen fermentation characteristics, microbial sequencing, and metabolomic analysis. All collected samples were meticulously preserved at a temperature of −80°C.

### Parameter determination

The ADG was calculated by subtracting the initial body weight from the final body weight and then dividing the result by the total number of days in the trial. Feed efficiency was evaluated based on the feed conversion ratio, which compares the average daily feed intake to the ADG. The serum biochemical indicators detected include creatine kinase for monitoring overall health, immunoglobulin G (IgG) for assessing immune function, and cortisol for evaluating stress conditions. Serum antioxidant indicators measured in this study included T-AOC, glutathione peroxidase (GSH-Px), superoxide dismutase (SOD), malondialdehyde (MDA), and reactive oxygen species (ROS). All assays were conducted strictly in accordance with the protocols provided by the corresponding assay kits from the Beijing Sinouk Institute of Biological Technology (Beijing, China).

The rumen pH value was promptly measured using a portable pH meter (Testo 206, Testo AG, Schwarzwald, Germany) as soon as the rumen contents were collected. The ammonia nitrogen (NH_3_-N) concentration was ascertained following the protocol established by Broderick and Kang [17], employing the phenol-hypochlorite colorimetric method. Meanwhile, the concentration of microbial crude protein (MCP) was evaluated using the Folin phenol method, which is an adaptation of Lowry’s assay, as detailed in the work of Makkar et al [18]. The detection of VFA was conducted using a gas chromatograph (Shimadzu GC2014, Shimadzu, Kyoto, Japan), which was fitted with a 30-meter capillary column (Rtx-Wax, 0.25 mm internal diameter×0.25 μm film thickness, Restek, Evry, France) and a flame ionization detector (FID-2014, Shimadzu). The injection volume was precisely set at 0.4 μL, and the injector temperature was maintained at 220°C. The oven temperature profile was as follows: starting at 110°C for 30 s, ramping up to 120°C over 3 min and holding for 4 min, then increasing to 150°C at a steady rate of 10°C/min. The split ratio was consistently set at 20:1, and the flow rate was held at 2.5 mL/min. Quantification was achieved using analytical standards sourced from Sigma-Aldrich (Merck KGaA, Darmstadt, Germany). The identity of each VFA was confirmed by comparing their relative retention times, and their concentrations were determined using the external standard method, as outlined in Wei et al [19]. The rumen microbial density was quantified by measuring the absorbance at 595 nm (OD_595nm_) following centrifugation at 10,000×g for 5 minutes [20]. The concentration of AI-2 was determined using the colorimetric assay developed by Wattanavanitchakorn et al [21]. Biofilm formation was assessed using the crystal violet staining technique, which involves methanol for fixation, crystal violet for staining, and ethanol for decolorization [20]. The extraction of extracellular polymeric substances was conducted according to the protocol described by Gu et al [20]. The quantity of exopolysaccharides was ascertained using the phenol-sulfuric acid method, with glucose as the calibration standard [22].

### DNA extraction, sequencing, and data analysis

DNA was extracted from twelve rumen fluid samples using a Bacterial DNA Kit (OMEGA, Omega Bio-Tek, Norcross, GA, USA), following a two-step protocol that incorporated bead-beating for effective initial lysis, as described in Paz et al [16]. The DNA’s integrity was confirmed through 1% agarose gel electrophoresis, while its concentration was determined using a spectrophotometer (NanoDrop 2000, Thermo Fisher Scientific, Wilmington, DE, USA). After quality control, twelve high-purity and high-quality DNA samples were sent to a biogenetic technology company (Allwegene, Beijing, China) for subsequent amplification and sequencing. The V3–V4 region of the 16S rRNA gene was targeted for amplification using primers 338F (5′-ACTCCTACGGGAGGCAGCAG-3′) and 806R (5′-GGACTACNNGGGTATCTAAT-3′) to reveal the diversity and community composition within the rumen fluid samples [23]. The PCR amplification protocol and parameter settings followed those of Qiu et al [24]. The PCR products were qualitatively checked using 1% agarose gel electrophoresis and purified using Agencourt AMPure XP Kits (Beckman Coulter, Brea, CA, USA). The purified products were then used for library construction. High-throughput sequencing was performed on the Illumina NextSeq 2000 platform using paired-end sequencing. The raw data generated from sequencing was deposited in the NCBI database under accession number PRJNA1158238.

The raw sequencing data underwent rigorous analysis using the Quantitative Insights Into Microbial Ecology (QIIME 2, version 2020.11, [25]) platform. The paired-end reads were deftly merged using the Fast Length Adjustment of Short reads (FLASH, version 1.2.11, [26]), with stringent parameters set for a maximum mismatch rate of 0.10 and a minimum overlap of 10 bp. Only sequences that complied with the following criteria were retained: a length between 200 and 500 base pairs, a quality score of no less than 20, an absence of ambiguous bases or chimeric sequences, and a match to the primer sequences and barcode tags. The sequences that cleared the quality control hurdles were denoised into amplicon sequence variants (ASV) using the Deblur algorithm integrated within QIIME 2. Each ASV was classified taxonomically using the Basic Local Alignment Search Tool (BLAST, v2.15.0, [27]) against the bacterial SILVA 138 database [28], and the e-value threshold was set to 1e^−5^. Alpha diversity indices were computed by means of QIIME 2 to provide a comprehensive characterization of the richness and evenness of the rumen bacterial community. To reveal the differences between the WT12 group and WT25 group, principal coordinates analysis (PCoA) based on Bray-Curtis were introduced using R software (version 4.0.2). An analysis of similarities (ANOSIM) was additionally performed to uncover the community structure similarities between the WT12 group and WT25 group, employing the vegan package within R software. The Phylogenetic Investigation of Communities by Reconstruction of Unobserved States (PICRUSt, version 2.5.2, [29]) was utilized to predict the metagenomic contributions of the identified microbial communities, thereby shedding light on the intrinsic ruminal functions associated with the bacterial microbiota.

### Metabolomics analysis

A total of 12 rumen fluid samples were sent to Beijing Allwegene Technology (Beijing, China) for non-targeted metabolomic analysis. To prepare the extraction solvent, methanol and acetonitrile were mixed in a 1:1 volume ratio. Then, 100 μL of rumen fluid was mixed with 400 μL of this solvent. After vortexing, ultrasonication, and centrifugation, the supernatant was collected as the original sample for metabolomics analysis. Additionally, an equal volume of the supernatant mixture was combined to form three samples serving as quality control samples. Analyses were performed using an ultra-high performance liquid chromatograph system (Acquity LC, Waters, Milford, CT, USA) coupled to an Orbitrap Exploris 120 mass spectrometer (Orbitrap, Thermo Fisher Scientific). Separation was performed on a Waters ACQUITY UPLC BEH Amide column (1.7 μm, 2.1×100 mm). Mobile phase A was 25 mM ammonium acetate and 25 mM ammonium hydroxide in water, and mobile phase B was 100% acetonitrile. The flow rate was set to 0.5 mL/min, while the injection volume was 2 μL. The Orbitrap Exploris 120 mass spectrometer was used to acquire MS/MS spectra in information-dependent acquisition (IDA) mode, with the acquisition software (Xcalibur, Thermo Fisher Scientific) controlling the process. In this mode, the acquisition software continuously evaluates the full scan MS spectrum. The electrospray ionization source conditions were set as follows: sheath gas flow rate 50 Arb, auxiliary gas flow rate 15 Arb, capillary temperature 320°C, full MS resolution 60,000, MS/MS resolution 15,000, collision energy stepped normalized collision energy 20/30/40, and spray voltage of 3.8 kV for positive mode and −3.4 kV for negative mode.

The initial raw data were transformed into mzXML format using the ProteoWizard software. Subsequently, these data were subjected to analysis through an in-house program developed in R, which leverages the XCMS framework for the detection, extraction, alignment, and integration of peaks. For the metabolite identification phase, a suite of R packages along with the comprehensive Human Metabolome Database were employed. During the analysis, metabolite features detected in more than 50% of the tested samples were removed from the data analysis, and missing values in the raw data were imputed with half of the minimum value. Additionally, internal standard normalization was employed, and features with a relative standard deviation greater than 30% were removed from further analysis. Three-dimensional result data involving peak quantity, sample names, and normalized peak areas were input into the R package MetaboAnalystR for principal component analysis and orthogonal partial least squares-discriminant analysis (OPLS-DA). To achieve a higher level of group separation and better understand the variables responsible for classification, supervised OPLS-DA was applied, and R^2^ and Q^2^ values were calculated. R^2^ represents the extent to which the variation is explained by the variables, while Q^2^ indicates the predictive ability of the variables. Subsequently, to check the robustness and predictive power of the OPLS-DA model, a permutation test was further conducted 200 times. To refine the analysis, the first principal component of variable importance in the projection (VIP) was introduced. The VIP value summarizes the contribution of each variable to the model. Metabolites with VIP>1, p<0.05 (student’s t-test), and fold change >1.5 or <0.67 were considered significantly differential metabolites. Furthermore, commercial databases including Kyoto Encyclopedia of Genes and Genomes and MetaboAnalyst were utilized to search for metabolic pathways of the metabolites.

### Statistics analysis

The Shapiro-Wilk test was used to evaluate the normality of the data distribution. Given that all data, except for the microbial data, adhered to the normality criteria, an independent samples t-test was conducted using SPSS software (version 20; IBM, Armonk, NY, USA) to assess the differences between the WT12 and WT25 groups. The threshold for statistical significance was set at p<0.05. For microbial data, the independent samples Mann-Whitney U test was applied, with a significance threshold of 0.05. Moreover, an LDA Effect Size (LEfSe) analysis was taken to pinpoint biomarkers within each group, with an LDA score threshold set at 3.0 to reduce false-positive rate.

The correlations between rumen and serum metabolites, microbial QS, and the microbiota were elucidated using a correlation matrix constructed in GraphPad Prism (version 10.1.2 [324], GraphPad Software, San Diego, CA, USA). Utilizing Spearman’s rank correlation coefficient (r) and p-values, a heatmap was generated to visualize these correlations. For inclusion in the heatmap, a threshold was set at Spearman’s r values greater than 0.70 or less than −0.70, along with a p-value of less than 0.05, ensuring significance. The heatmap itself was crafted using the same software mentioned above.

## RESULTS

### Growth performance

The effect of drinking water temperature on the growth performance of Hu sheep is shown in Table 2. The WT25 group exhibited higher weight gain and ADG throughout the trial period, and the feed to gain ratio was lower compared with the WT12 group (p<0.05). However, no significant variation was observed in the average dry matter intake between these two groups (p>0.05).

### Serum biochemical, immune capacity, and antioxidant indicators

As shown in Table 3, drinking water temperature had no significant effect on the concentration of creatine kinase (p>0.05). The WT25 group had higher serum IgG levels compared to the WT12 group, while cortisol levels were higher in the WT12 group (p<0.05). The WT25 group exhibited higher levels of T-AOC, GSH-Px, and SOD than the WT12 group, whereas the levels of MDA, ROS, and the oxidative stress index (OSI) were higher in the WT12 group than in the WT25 group (p<0.05).

### Rumen fermentation characteristics

The effects of drinking water temperature on rumen fermentation characteristics are listed in Table 4. The WT25 group exhibited a markedly higher MCP concentration compared to the WT12 group, with a contrasting trend observed in NH_3_-N levels (p<0.05). Despite a numerical rise in the levels of individual VFA, the total VFA, and the branched-chain VFA within the WT25 group, these increases failed to reach statistical significance (p>0.05). Echoing the pattern observed with VFA concentrations, the proportions of individual and branched-chain VFA did not differ significantly between the two groups (p>0.05).

### Rumen microbial quorum sensing

**Figure 1:**
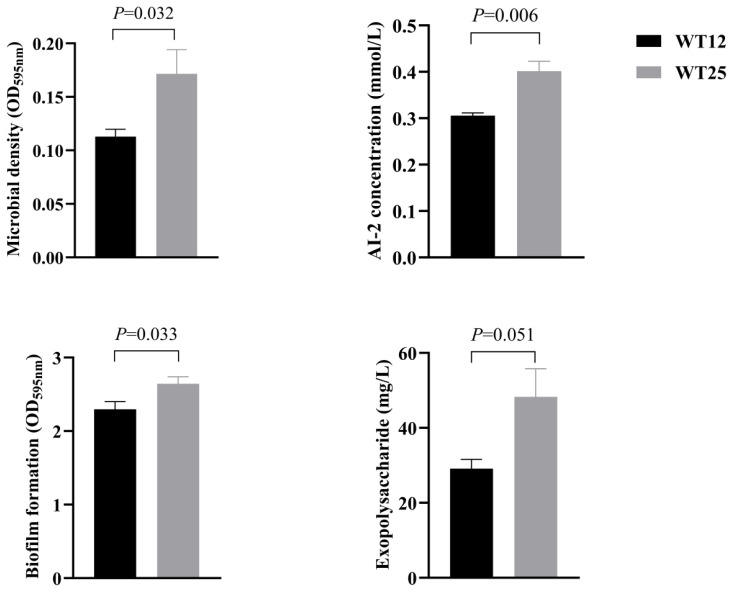
Effects of drinking water temperatures of 12°C (WT12) or 25°C (WT25) on quorum sensing in rumen bacteria of Hu sheep.

Figure 1 demonstrates the effects of drinking water temperature on the QS in rumen bacteria of Hu sheep. The WT25 group exhibited higher microbial density, AI-2 signaling molecule concentration, and biofilm formation in rumen fluid compared to the WT12 group (p<0.05).

### Rumen microbial diversity and community composition

**Figure 2:**
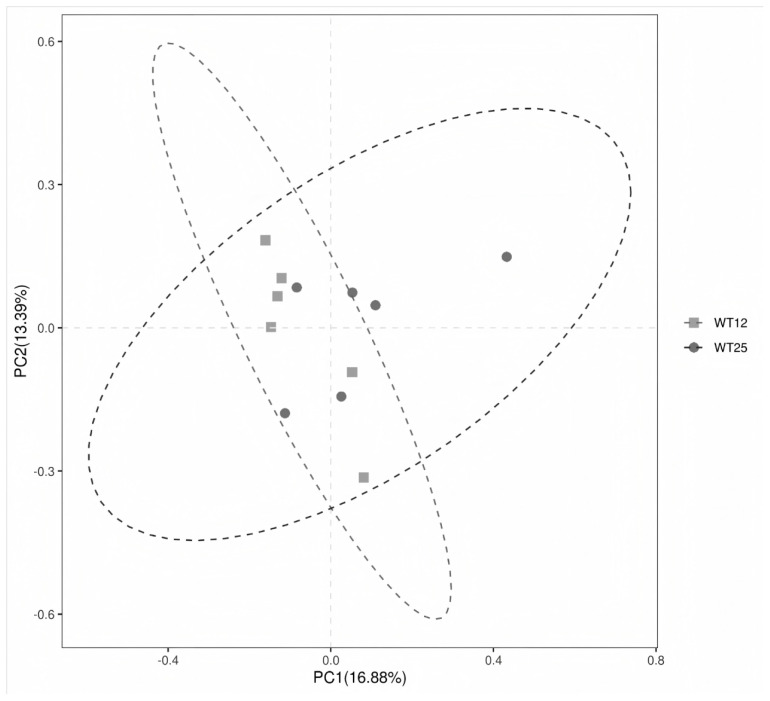
Principal coordinates analysis (PCoA) of rumen bacteria in response to drinking water temperatures of 12°C (WT12) or 25°C (WT25).

Effects of drinking water temperature on the alpha-diversity of rumen microorganisms in Hu sheep are detailed in Table 5. Drinking water temperature did not affect the richness and evenness of the rumen microbial community (p>0.05). At the phylum level (Table 6), Bacteroidota and Firmicutes were the dominant phyla, represented 51.26% and 43.86%, respectively. Moreover, the relative abundances of phyla with more than 0.1% were not affected by drinking water temperature (p>0.05). At the genus level (Table 7), the WT25 group showed lower relative abundances of *Muribaculum* and *Clostridia UCG-014* as compared to the WT12 group (p<0.05). Additionally, PCoA (Figure 2) showed clear intersections, and ANOSIM also showed no significant differences in rumen microbial communities between the WT12 and WT25 groups (p = 0.611). Among the methanogenic archaea, the most abundant genus identified in this study was *Methanobrevibacter*. Notably, there was no significant difference in the relative abundance of *Methanobrevibacter* between the WT12 and WT25 groups (p>0.05).

**Figure 3:**
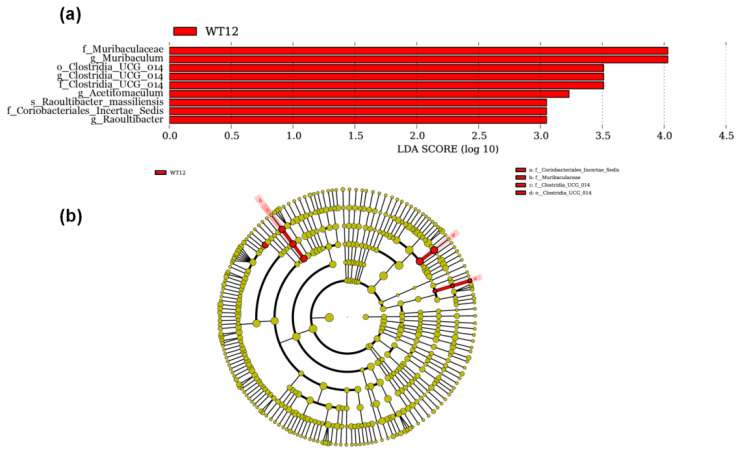
Effect of drinking water temperatures of 12°C (WT12) or 25°C (WT25) on discriminative bacterial communities across various taxonomic levels is illustrated using (a) linear discriminant analysis and (b) cladogram. Species with no significant differences are uniformly colored yellow, while differential species biomarkers are colored according to their respective groups.

A biomarker analysis was conducted to identify differentially abundant species across the WT12 and WT25 groups at various taxonomic levels, as illustrated in the form of a LefSe plot (LDA effect size bar graph, Figure 3A) and a cladogram (Figure 3B). The LDA effect size bar graph indicated that a total of nine species were identified with an LDA score exceeding 3.0 and a significance level of p<0.05. The biomarkers in the WT12 group exhibited significant discriminative power, including f_Muribaculaceae, g_*Muribaculum*, o_Clostridia UCG-014, f_Clostridia UCG-014, g_*Clostridia UCG-014*, *Acetitomaculum*, *Raoultibacter massiliensis*, Coriobacteriales Incertae Sedis, *Raoultibacter*. The results of the cladogram are consistent with the LefSe analysis.

This study identified a total of 19 gene families in the samples, with 8 of these having relative abundances greater than 5% (Table 8). Notably, the gene families linked to the metabolism of other amino acids in the WT25 group exhibited a marked increase in comparison to the WT12 group (p<0.05). Conversely, the lipid metabolism category displayed a significant decrease (p<0.05).

### Rumen metabolome profile

As shown in Table 9, a total of 12 metabolites were identified as differentially abundant between the WT25 and WT12 groups, meeting the criteria of a VIP score greater than 1, a p-value less than 0.05, and a fold change greater than 1.5 or less than 0.67. Among these 12 differential metabolites, 8 were increased in the WT25 group, and the other 4 decreased. The decreased metabolites are mainly found in carboxylic acids and derivatives (orlistat, Lys-Gln, and L-norleucine) and steroids and steroid derivatives (17alpha-nandrolone). The increased metabolites are primarily distributed across isoindoles/indoles and derivatives (lenalidomide, gelsemine, and indoleacrylic acid), imidazopyrimidines (trans-zeatin), phenylpropanoic acids (phenyllactic acid), organic phosphoric acids and derivatives (perifosine), benzimidazoles (5,6-dimethylbenzimidazole), and organonitrogen compounds (L-histidinol).

### Correlations between rumen fermentation characteristics, metabolites, serum antioxidant capacity, and rumen microbiota

**Figure 4:**
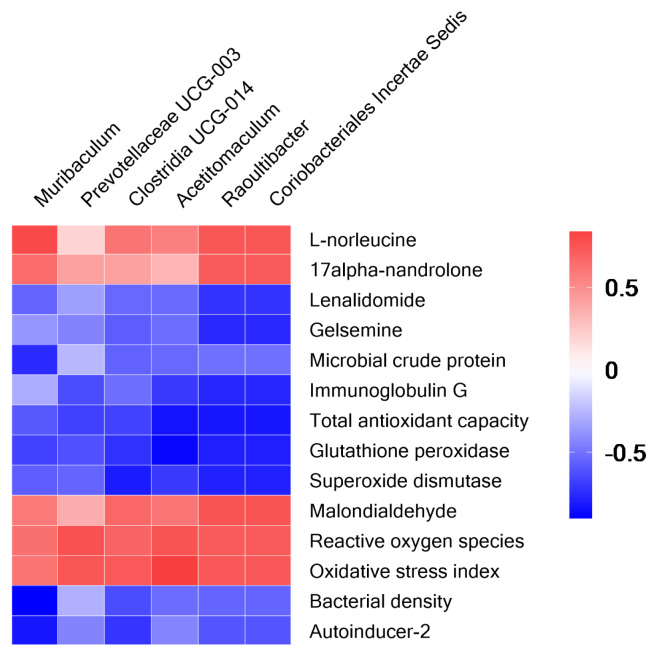
Spearman correlation analysis between rumen microbiome and metabolite, quorum sensing, serum immunity, and antioxidant capacity. Colors indicate positive (red, closer to 1) or negative (blue, closer to −1) correlations.

As shown in Figure 4, five genera and one family were observed to be highly correlated with at least one of the parameters detected in this study. *Raoultibacter* and Coriobacteriales Incertae Sedis were positively correlated with L-norleucine, 17alpha-nandrolone, NH_3_-N, MDA, ROS, and OSI, and negatively correlated with lenalidomide, gelsemine, IgG, T-AOC, GSH-Px, and SOD. *Clostridia UCG-014* and *Acetitomaculum* were observed to have a negative association with GSH-Px and SOD, as well as a positive association with OSI. *Prevotellaceae UCG-003* was found to have a positive association with ROS and OSI. Moreover, *Muribaculum* was negatively correlated with MCP, bacterial density, and AI-2. The concentration of AI-2 was negatively correlated with the relative abundance of *Clostridia UCG-014*.

## DISCUSSION

### Effect of drinking water temperature on growth performance and antioxidant capacity of Hu sheep

The consumption of cold water by ruminants can lead to a decrease in rumen temperature, which subsequently impairs microbial activity and the secretion of digestive enzymes, adversely affecting growth performance [6]. The rumen’s microenvironment is particularly sensitive to temperature fluctuations, and a reduction in temperature can significantly slow the metabolic processes of microbes crucial for breaking down feed into assimilable nutrients [30]. The resulting diminished microbial activity leads to less efficient fermentation, a process essential for converting fibrous plant materials into usable energy and amino acids for the host [31]. Consequently, the animal’s ability to absorb dietary nutrients is negatively impacted, leading to reduced growth and overall performance [7]. In this study, drinking heated water in winter increased the ADG and feed efficiency of sheep, which is consistent with the aforementioned reports in beef and dairy cattle [6,7,31], possibly due to the higher water temperature being more suitable for the growth and reproduction of rumen microorganisms, as well as for the activity of more digestive enzymes for fermentation.

IgG is a cornerstone of the immune system, providing long-term protection against a broad spectrum of pathogens. It activates the complement system, leading to the lysis and destruction of pathogens, and enhances the process of opsonization, which allows for more effective immune cell engagement and destruction of microbes [32]. A higher level of IgG was observed in the WT25 group, indicating that consuming heated water in winter can boost the immune system of sheep. Cortisol, a glucocorticoid hormone, is released in response to stressors and plays a crucial role in the body’s stress response. Higher concentrations of cortisol are associated with greater stress loads, which can adversely affect health, growth, and immune function [33]. In this study, the WT25 group showed a decrease in cortisol levels, possibly due to the reduced stress on the rumen from drinking heated water compared to cold water. The serum T-AOC, GSH-Px, and SOD are essential for maintaining redox balance in ruminants. T-AOC reflects the body’s overall ability to neutralize free radicals, which can cause cellular damage if left unchecked [34]. GSH-Px plays a critical role in the detoxification process by reducing lipid peroxides, thereby protecting cells from oxidative damage and supporting the immune system [35]. SOD serves as the first line of defense against superoxide radicals, converting them into less reactive species and thus preventing oxidative stress [36]. On the other hand, MDA and ROS are indicators of oxidative stress and can be harmful to the organism [37]. High levels of MDA and ROS can lead to cellular and tissue injury, inflammation, and disease states [37,38]. This study found that the WT25 group exhibited increased levels of T-AOC, GSH-Px, and SOD, while the levels of MDA and ROS were reduced. Additionally, the OSI also demonstrated a decrease. These findings indicate that the antioxidant capacity of the Hu sheep was enhanced and the degree of oxidative stress was reduced after drinking heated water. This is consistent with the lower stress levels indicated by the IgG and cortisol responses previously mentioned. The results suggest that drinking heated water in winter can mitigate oxidative stress, bolster antioxidant and immune capabilities, and foster improved growth performance in Hu sheep.

### Effect of drinking water temperature on rumen fermentation characteristics and rumen metabolites of Hu sheep

Rumen fermentation is pivotal for ruminants as it facilitates the conversion of fibrous plant material into absorbable nutrients and energy [39]. This microbial-driven process is essential for animal nutrition and health, influencing feed efficiency and productivity. Rumen NH_3_-N serves as an essential nitrogen source for the synthesis of MCP, providing the host animal with vital amino acids necessary for its growth and development [39]. The WT25 group exhibited higher levels of MCP and a lower concentration of NH_3_-N. This could be attributed to the similar amount of dietary nitrogen in the current feeding pattern, which allows for a greater conversion of NH_3_-N into MCP through microbial activity. This phenomenon occurs because the NH_3_-N level reflects the dynamic equilibrium between the degradation products of dietary protein and the synthesis of MCP as a nitrogen source [40].

The rumen differential metabolites offer a more nuanced understanding of the enhanced immune capabilities and superior growth performance observed in the WT25 group. Lenalidomide not only strengthens the functionality of natural killer cells but also modulates the immune system by inhibiting the nuclear factor κB pathway, reducing the levels of tumor necrosis factor *α* and cyclooxygenase-2, and dampening T-cell co-stimulation [41]. Gelsemine, an alkaloid derived from Gelsemium species, exhibits anti-inflammatory properties by suppressing the overexpression of pro-inflammatory cytokines [42]. This study identified an upregulation of lenalidomide and gelsemine in the WT25 group, which correlates with their roles in immunomodulation and inflammation reduction, as supported by the elevated IgG levels observed within this group. Indoleacrylic acid, derived from tryptophan, and L-histidinol, a derivative of histidine, both positively contribute to the organism’s amino acid composition, intestinal health, and immune function [43]. Our study found elevated levels of these compounds in the WT25 group, indicating that increasing the temperature of drinking water in winter can enhance the immune capabilities of sheep. This enhancement is further supported by the higher levels of IgG and antioxidant activity observed in the serum. Trans-zeatin and its derivatives are capable of modulating key cellular signaling pathways, including Nrf2 and PI3K/Akt, which contribute to their antioxidant, anti-aging, cell-protective, and anti-inflammatory effects [44]. Perifosine, functioning as an Akt inhibitor, has demonstrated promise in the treatment of a range of cancers [45]. The elevated levels of trans-zeatin and perifosine observed in the WT25 group imply enhanced antioxidant and immune capabilities within this cohort. Phenyllactic acid is known to enhance animal growth and bolster the immune system, as well as decrease the count of *Escherichia coli* in the large intestine [46]. Research in broiler chicks has also shown that it can increase the count of blood cells related to immunity [47]. Similarly, our study observed higher concentrations of phenyllactic acid in the WT25 group, which had stronger immune capabilities, suggesting that increasing the temperature of drinking water during winter can positively affect the immune system. 5,6-dimethylbenzimidazole is a precursor of vitamin B12 that plays a critical role in enhancing the synthesis of vitamin B12 in the rumen [48], and vitamin B12 is known to strengthen the immune system and support gastrointestinal health. Our study found elevated levels of 5,6-dimethylbenzimidazole in the rumen fluid of the WT25 group, suggesting that increasing the drinking water temperature may enhance immune function and gastrointestinal health. Orlistat can reduce fat absorption by inhibiting gastrointestinal lipase, which in turn leads to a decrease in the absorption of fat-soluble vitamins, potentially adversely affecting the health of animals [49]. Previous study found that high doses of Lys-Gln reduced the efficiency of amino acid utilization in the jejunum and suppress protein synthesis through the GCN2/eIF2α/ATF4 signaling pathway [50]. Additionally, L-norleucine, an isomer of leucine, accelerates the catabolism of endogenous amino acids and affects both their synthesis and that of proteins [51]. The upregulation of orlistat, Lys-Gln, and L-norleucine in the WT12 group suggests that consuming cold water may reduce the efficiency of amino acid and vitamin utilization, thereby impairing growth performance. 17alpha-nandrolone is an anabolic steroid, and excessive use can lead to impaired kidney and liver function, exacerbate hormonal activity, and disrupt the balance of oxidants/antioxidants [52]. This experiment found that the 17alpha-nandrolone levels in the WT12 group were high, which corresponds with the higher levels of OSI, ROS, and MDA in the serum of this group, and also suggests that drinking cold water may cause organ damage.

### Effect of drinking water temperature on rumen microbial quorum sensing, diversity and community composition of Hu sheep

Bacterial density plays a critical role in QS, as it triggers the activation of collective behaviors that are beneficial to the bacterial community. At elevated cell densities, QS facilitates synchronized gene expression and the coordinated execution of group actions, such as bioluminescence and biofilm formation [53]. These actions would be inefficient or wasteful if performed by individual bacteria in isolation. Higher microbial density and biofilm formation were observed in the rumen fluid of sheep in the WT25 group, indicating that drinking heated water in the cold season could activate the collective behaviors of rumen microbes, and form more biofilm to resist cold stimulation. This hypothesis is further supported by the higher concentration of AI-2 signaling molecules found in the WT25 group. AI-2, being the most common LuxS/AI-2 QS chemical signal among rumen microbes, serves as a communication signal that facilitates the implementation of collective behavior by microorganisms [15].

Rumen microbial alpha-diversity plays a pivotal role in maintaining the stability and functionality of the rumen ecosystem. It reflects the richness and evenness of microbial species, which is crucial for the efficient digestion of feed and nutrient absorption [54]. No significant differences were found between the WT12 and WT25 groups, which is consistent with the findings of He et al [6], who reported no significant differences in rumen microbial alpha-diversity in beef cattle when drinking water temperatures were 4.4°C and 18.6°C. This suggests that the temperature of drinking water has little impact on rumen microbial diversity, possibly because the rumen microbiota possesses certain regulatory capabilities, such as the ability to form biofilms to collectively resist external adversity, as mentioned earlier. Muribaculaceae is recognized for its association with health benefits, primarily due to its cross-feeding relationship with probiotics like *Bifidobacterium* and *Lactobacillus*, which contributes to a synergistic role within the gut ecosystem [55]. However, there is also evidence suggesting that an overabundance of Muribaculaceae could lead to inflammation [56]. The WT25 group exhibited a reduced relative abundance of *Muribaculum* and enhanced immune function, a finding that aligns with subsequent research suggesting potential adverse effects of *Muribaculum* on host health. Nonetheless, further investigation is warranted to elucidate the precise role of *Muribaculum* in animal health. *Clostridia UCG-014* has been associated with disruptions in microbial community balance and the onset of metabolic disorders. Wang et al [57] reported that the abundance of *Clostridia UCG-014* increases in response to gut microbiota and serum metabolic perturbations induced by crotonis fructus. It was also reported that an increase in the abundance of *Clostridia UCG-014* may be associated with inflammatory responses and cardiac damage [58]. This study found that the relative abundance of *Clostridia UCG-014* in the WT25 group was lower than in the WT12 group, indicating that drinking heated water in winter can reduce immune inflammation. This finding corresponds with the higher levels of IgG and antioxidant capacity, as well as lower level of cortisol, observed in the serum of the WT25 group. *Methanobrevibacter* is recognized as one of the predominant methanogenic archaeal genera within the rumen of ruminants. It plays a pivotal role in the production of methane by utilizing hydrogen and carbon dioxide. This metabolic pathway not only results in an energy loss for the host animals but also contributes to the intensification of the greenhouse effect [59]. In this study, there was no significant difference in the relative abundance of *Methanobrevibacter* between the WT12 and WT25 groups. This finding indicates that altering the drinking water temperature does not affect methanogen populations. Such a result could be attributed to the robust buffering capabilities of the rumen environment and the inherent adaptability of these methanogenic archaea to varying conditions.

Coriobacteriales Incertae Sedis is a group of bacteria within the Coriobacteriia class, the specific classification of which has not yet been determined. This taxonomic group has been identified in the human gut microbiome, however, their ecological range and genomic diversity are not yet fully understood [60]. The study found a higher abundance of this family in the WT12 group, suggesting a potential association with the occurrence of inflammation. *Raoultibacte*r, along with its type species *Raoultibacter massiliensis*, may affect the levels of T cells in the gut, potentially impacting the host’s immune response and inflammatory status [61]. A higher relative abundance of these bacteria was observed in the WT12 group, further supporting their role in reducing immunity and promoting inflammatory responses. Li et al [62] found that high-starch diets stimulated the rapid production of VFA and lactic acid, leading to a rapid decrease in rumen pH. This decrease can trigger chronic inflammation and dysfunction of the rumen papillae. During this process, the relative abundance of *Acetitomaculum* increases. Our study also found that the immune and antioxidant capabilities are lower in the WT12 group when compared to the WT25 group, along with a higher relative abundance of *Acetitomaculum*, further confirming the role of the genus *Acetitomaculum* in inflammatory responses.

### Correlations between rumen fermentation characteristics, metabolites, serum antioxidant capacity, and rumen microbiota

Correlation analysis offers a new perspective on the relationship between rumen microbes and rumen metabolites, indirectly shedding light on the potential functions of these metabolites and microbes. As mentioned above, *Raoultibacter* is reported to affect the host’s immune response [61]. This study revealed that its relative abundance is negatively associated with antioxidant capacity (T-AOC, GSH-Px, and SOD), immune response (IgG), and the production of beneficial metabolites such as lenalidomide and gelsemine. These findings further confirm the negative function of this bacterium in impaired immunity and induced oxidative stress. Similarly, Coriobacteriales Incertae Sedis showed similar associations with these antioxidant properties and rumen metabolites, suggesting its involvement in the occurrence of inflammation. Both *Clostridia UCG-014* and *Acetitomaculum* have been shown to be common in cases of metabolic disorders or inflammatory responses [57,62], which well explains the positive association with OSI and the negative association with GSH-Px and SOD. Moreover, *Clostridia UCG-014* is also reported to be highly abundant in cases of microbiota perturbations [57], while the AI-2 signaling molecule in QS plays an important role in maintaining the stability of rumen microbial communities [63]. These findings well explain the negative correlation between *Clostridia UCG-014* and AI-2 signaling molecules. Previous studies found that *Prevotellaceae UCG-003* may be linked to the accumulation of metabolic products such as acetate and methane in the rumen, potentially leading to decreased rumen fermentation and negatively impacting the growth performance of goats [64]. Additionally, it was suggested that *Prevotellaceae UCG-003* may play a beneficial role in the immune-inflammatory system [65]. This study observed a positive correlation between *Prevotellaceae UCG-003* and ROS and OSI, suggesting that this bacterium may also be associated with oxidative stress. Muribaculaceae was previously widely regarded as a beneficial microorganism in the gut; however, an overabundance of Muribaculaceae may lead to inflammation [55,56]. This study found a negative correlation between *Muribaculum* and MCP concentration, bacterial density, and AI-2 signaling molecules in rumen fluid. This suggests that *Muribaculum* might influence MCP synthesis by inhibiting QS among rumen microorganisms, which could ultimately affect the growth performance of ruminants. The correlation analysis presented above highlight significant relationships between the rumen microbiota and the host’s immune and antioxidant capacities. Additionally, it provides valuable insights into the roles of specific microbial genera in QS mechanisms within the rumen microbial community.

## CONCLUSION

Taken together, drinking heated water in winter improved growth performance, serum immune function, and antioxidant capacity, while also partially affecting rumen fermentation characteristics, bacterial composition, QS, and metabolomic profiles of Hu sheep. Compared to the WT12 group, the WT25 group exhibited higher ADG, serum IgG, T-AOC, GSH-Px, and SOD, along with lower feed-to-gain ratio, serum cortisol, MDA, ROS, and OSI. The concentrations of MCP, microbial density, AI-2 signaling molecule, and biofilm formation were higher in the WT25 group, while the NH_3_-N concentration was lower compared to the WT12 group. The relative abundances of *Muribaculum* and *Clostridia UCG-014*, as well as the predicted metabolic pathways related to lipid metabolism, were higher in the WT12 group, whereas the metabolism of other amino acids showed increased abundances in the WT25 group. Metabolomics analysis identified 12 differential metabolites, four of which were correlated with *Muribaculum*, *Raoultibacter*, and Coriobacteriales Incertae Sedis. This research not only provides strategies for optimizing environmental factors in the precision feeding of ruminants but also offers new insights for a comprehensive exploration of how rumen microbial QS respond to environmental stressors.

## Figures and Tables

**Table 1:** Dietary ingredient composition and nutritional content on a dry matter basis

Ingredient	Proportion (%)	Nutritional content	Value
Corn	24.04	Metabolizable energy (MJ/kg)	9.53
Soybean	15.72	Crude protein (g/kg)	130.85
Wheat bran	6.49	Ether extract (g/kg)	23.61
Wheat straw	51.00	Neutral detergent fiber (g/kg)	454.63
Calcium hydrogen phosphate	0.75	Acid detergent fiber (g/kg)	268.11
Limestone	0.50		
Salt	0.50		
Premix^1)^	1.00		

1) Premix provided the following per kg of dry matter (DM): 1,400 mg of Fe, 1,200 mg of Zn, 250 mg of Cu, 900 mg of Mn, 100,000 IU of vitamin A, 27,000 IU of vitamin D3, and 800 IU of vitamin E.

**Table 2:** Effect of drinking water temperature on growth performance of Hu sheep

Item	WT12^1)^	WT25^2)^	SEM	p-value
Initial body weight (kg)	27.73	27.67	0.697	0.952
Body weight gain (kg)	7.08	9.35	0.518	0.033
Average daily gain (g/d)	157.22	207.86	11.489	0.033
Average dry matter intake (g/d)	933.63	926.22	15.369	0.747
Feed to gain ratio^3)^	5.96	4.68	0.297	0.023

1) WT12 represents the drinking water temperature of 12°C.

2) WT25 represents the drinking water temperature of 25°C.

3) Feed to gain ratio is calculated as dry matter intake divided by average daily gain.

SEM, standard error of the mean.

**Table 3:** Effect of drinking water temperature on serum biochemical, immune capacity, and antioxidant indicators of Hu sheep

Item	WT12^1)^	WT25^2)^	SEM	p-value
Creatine kinase (U/L)	199.65	174.23	24.840	0.506
Immunoglobulin G (g/L)	13.16	18.52	1.138	0.034
Cortisol (ng/mL)	51.37	41.87	2.517	0.024
Total antioxidant capacity (U/mL)	29.08	38.55	0.658	<0.001
Glutathione peroxidase (U/mL)	260.95	316.24	6.689	<0.001
Superoxide dismutase (U/mL)	66.17	79.90	1.102	<0.001
Malondialdehyde (nmol/mL)	4.43	3.62	0.127	0.001
Reactive oxygen species (U/mL)	311.45	207.88	13.809	<0.001
Oxidative stress index^3)^	10.74	5.43	0.525	<0.001

1) WT12 represents the drinking water temperature of 12°C.

2) WT25 represents the drinking water temperature of 25°C.

3) Oxidative stress index is calculated as reactive oxygen species divided by total antioxidant capacity.

SEM, standard error of the mean.

**Table 4:** Effect of drinking water temperature on rumen fermentation characteristics of Hu sheep

Item	WT12^1)^	WT25^2)^	SEM	p-value
pH value	6.91	6.82	0.028	0.071
Ammonia nitrogen (mg/dL)	11.44	9.14	0.664	0.036
Microbial crude protein (mg/L)	221.08	285.37	15.858	0.017
VFA Concentration (mmol/L)				
Acetate	24.06	25.56	1.255	0.423
Propionate	5.01	5.18	0.324	0.716
Isobutyrate	0.49	0.54	0.028	0.242
Butyrate	3.86	4.23	0.192	0.213
Isovalerate	0.86	0.95	0.059	0.352
Valerate	0.36	0.43	0.021	0.056
Total volatile fatty acids	34.65	36.90	1.695	0.376
Branched-chain volatile fatty acids	1.72	1.92	0.107	0.216
VFA Proportion (%)				
Acetate	69.41	69.25	0.601	0.856
Propionate	14.45	14.02	0.452	0.548
Acetate to propionate ratio	4.86	4.95	0.183	0.744
Isobutyrate	1.44	1.48	0.083	0.699
Butyrate	11.13	11.51	0.302	0.434
Isovalerate	2.52	2.57	0.176	0.841
Valerate	1.06	1.17	0.053	0.160
Branched-chain volatile fatty acids	5.01	5.23	0.307	0.632

1) WT12 represents the drinking water temperature of 12°C.

2) WT25 represents the drinking water temperature of 25°C.

SEM, standard error of the mean.

**Table 5:** Effect of drinking water temperature on rumen bacterial alpha-diversity of Hu sheep

Item	WT12^1)^	WT25^2)^	SEM	p-value
Chao1	2,998.54	2,865.69	75.822	0.240
Observed species	2,715.35	2,524.08	84.496	0.132
Phylogenetic diversity whole tree	94.09	93.22	1.558	0.699
Shannon index	9.94	9.32	0.186	0.093
Simpson index	0.9972	0.9870	0.004	0.065

1) WT12 represents the drinking water temperature of 12°C.

2) WT25 represents the drinking water temperature of 25°C.

SEM, standard error of the mean.

**Table 6:** Effect of drinking water temperature on the relative abundance of rumen bacterial composition at phylum level of Hu sheep

Phylum name	WT12^1)^	WT25^2)^	SEM	p-value
Bacteroidota	50.39	52.13	2.068	0.485
Firmicutes	45.32	42.40	1.901	0.310
Verrucomicrobiota	1.14	1.43	0.236	0.699
Patescibacteria	0.89	0.79	0.143	0.699
Proteobacteria	0.73	0.91	0.106	0.180
Synergistota	0.13	0.85	0.207	0.065
Desulfobacterota	0.48	0.46	0.055	0.589
Actinobacteriota	0.34	0.24	0.038	0.180
Euryarchaeota	0.18	0.15	0.033	0.818
Spirochaetota	0.18	0.11	0.026	0.180
Elusimicrobiota	0.01	0.24	0.096	0.132
Cyanobacteria	0.08	0.12	0.035	0.485

1) WT12 represents the drinking water temperature of 12°C.

2) WT25 represents the drinking water temperature of 25°C.

SEM, standard error of the mean.

**Table 7:** Effect of drinking water temperature on the relative abundance of rumen bacterial composition at genus level of Hu sheep

Genus name	WT12^1)^	WT25^2)^	SEM	p-value
*Prevotella*	21.26	27.54	2.418	0.180
*Rikenellaceae RC9 gut group*	6.55	5.97	0.721	0.937
*Prevotellaceae F082*	6.21	5.56	0.626	0.589
*Christensenellaceae R-7 group*	5.61	4.81	0.580	0.394
*Muribaculum*	4.87	3.42	0.384	0.009
*Ruminococcaceae NK4A214 group*	3.98	3.08	0.432	0.180
*Prevotellaceae UCG-003*	3.79	2.99	0.301	0.093
*Succiniclasticum*	3.32	3.39	0.331	1.000
*Prevotellaceae UCG-001*	2.91	2.30	0.282	0.589
*Butyrivibrio*	2.34	2.63	0.298	0.589
*Mollicutes RF39*	1.85	1.55	0.139	0.132
*Lachnospiraceae XPB1014 group*	1.54	1.82	0.378	0.937
*Lachnospiraceae NK3A20 group*	1.78	1.26	0.221	0.093
*Ruminococcus*	1.70	1.20	0.208	0.132
*Veillonellaceae UCG-001*	1.33	1.51	0.378	0.699
*Eubacterium coprostanoligenes group*	1.43	1.38	0.153	0.699
*Bacteroidales RF16 group*	1.28	1.45	0.288	0.699
*Clostridia UCG-014*	1.62	0.90	0.144	0.015
*Firmicutes WCHB1-41*	1.13	1.38	0.236	0.818
*Bacteroidaceae p-251-o5*	1.33	0.73	0.291	0.240
*Ruminococcaceae UCG-010*	1.05	0.97	0.116	0.485

1) WT12 represents the drinking water temperature of 12°C.

2) WT25 represents the drinking water temperature of 25°C.

SEM, standard error of the mean.

**Table 8:** Effect of drinking water temperature on the relative abundance of the predicted metabolic pathways in the ruminal bacterial microbiome of Hu sheep

Item	WT12^1)^	WT25^2)^	SEM	p-value
Carbohydrate metabolism	14.15	14.09	0.046	0.310
Metabolism of cofactors and vitamins	13.94	14.00	0.084	1.000
Amino acid metabolism	13.40	13.30	0.052	0.240
Metabolism of terpenoids and polyketides	9.61	9.32	0.094	0.065
Metabolism of other amino acids	6.55	6.84	0.055	0.026
Replication and repair	6.60	6.53	0.019	0.065
Energy metabolism	5.40	5.35	0.018	0.065
Glycan biosynthesis and metabolism	5.13	5.25	0.087	0.589
Lipid metabolism	4.43	4.23	0.071	0.026
Translation	3.69	3.64	0.017	0.132
Folding, sorting and degradation	3.17	3.11	0.022	0.180
Biosynthesis of other secondary metabolites	2.39	2.41	0.023	1.000
Nucleotide metabolism	2.23	2.22	0.006	0.310
Cell motility	2.18	2.14	0.086	0.818
Cell growth and death	1.72	1.72	0.008	1.000
Membrane transport	1.50	1.48	0.023	0.394
Xenobiotics biodegradation and metabolism	1.18	1.67	0.322	0.818
Transcription	1.22	1.19	0.011	0.180
Signal transduction	0.30	0.29	0.007	0.310

1) WT12 represents the drinking water temperature of 12°C.

2) WT25 represents the drinking water temperature of 25°C.

SEM, standard error of the mean.

**Table 9:** Differential rumen metabolites between drinking water temperature of 25°C (WT25) and drinking water temperature of 12°C (WT12)

Pathway	Metabolites	Mass	Formula	VIP	p-value	Fold change	Trend
Isoindoles and derivatives	Lenalidomide	259.10	C_13_H_13_N_3_O_3_	1.67	0.032	1.52	Up
Indoles and derivatives	Gelsemine	322.40	C_20_H_22_N_2_O_2_	1.73	0.043	1.84	Up
Indoles and derivatives	Indoleacrylic acid	187.06	C_11_H_9_NO_2_	1.76	0.035	2.28	Up
Imidazopyrimidines	Trans-zeatin	219.11	C_10_H_13_N_5_O	2.32	0.003	1.53	Up
Phenylpropanoic acids	Phenyllactic acid	166.06	C_9_H_10_O_3_	1.41	0.043	1.65	Up
Organic phosphoric acids and derivatives	Perifosine	461.36	C_25_H_52_NO_4_P	2.02	0.010	1.67	Up
Benzimidazoles	5,6-dimethylbenzimidazole	146.08	C_9_H_10_N_2_	1.71	0.043	1.93	Up
Organonitrogen compounds	L-histidinol	141.09	C_6_H_11_N_3_O	1.89	0.017	1.98	Up
Carboxylic acids and derivatives	Orlistat	495.39	C_29_H_53_NO_5_	1.82	0.017	0.28	Down
Carboxylic acids and derivatives	Lys-Gln	274.16	C_11_H_22_N_4_O_4_	1.82	0.040	0.65	Down
Carboxylic acids and derivatives	L-norleucine	131.09	C_6_H_13_NO_2_	2.22	0.001	0.66	Down
Steroids and steroid derivatives	17alpha-nandrolone	274.40	C_18_H_26_O_2_	1.85	0.035	0.43	Down

VIP, variable importance in the projection.
